# Self-reported hearing difficulties and speech-in-noise test performance - what can we find behind a “normal” audiogram?

**DOI:** 10.1590/2317-1782/20232022111p

**Published:** 2023-11-27

**Authors:** Pamela Papile Lunardelo, Laura Caetano Meneghelli, Sthella Zanchetta

**Affiliations:** 1 Faculdade de Filosofia, Ciências e Letras de Ribeirão Preto - FFCLRP, Universidade de São Paulo - USP - Ribeirão Preto (SP), Brasil.; 2 Faculdade de Medicina de Ribeirão Preto - FMRP, Universidade de São Paulo - USP - Ribeirão Preto (SP), Brasil.

**Keywords:** Auditory Perception, Speech Perception, Signal-Noise Ratio, Surveys and Questionnaires, Adult

## Abstract

**Purpose:**

To investigate complaints of difficulty understanding speech in the presence of noise in subjects without hearing loss and their performance on a speech-in-noise test.

**Methods:**

Thirty-nine subjects aged 18 to 59 years and 11 months were divided into four groups according to their decade of life. They underwent audiometry, tympanometry, auditory processing tests, the Mini-Mental State Examination, a self-report on auditory perception combined with the Amsterdam Inventory for Auditory Disability and Handicap (Pt-AIADH), and a sentence test in silence and in noise.

**Results:**

All groups scored high on the Pt-AIADH domains, with the highest average score obtained for the noise intelligibility domain. There were differences between G18 vs. G40, G18 vs. G50, and G30 vs. G50 for auditory self-perception in noise intelligibility, and differences between the youngest and all other groups on the speech-in-noise test in particular, with a lower signal-to-noise ratio for older adults. We also identified a moderate and significant correlation between intelligibility in noise and the speech-in-noise test.

**Conclusion:**

Normal hearers of all age groups complained of intelligibility in noise. We found that the higher an individual’s auditory difficulty in this domain, the worse their performance on the speech-in-noise test; this is especially true for middle-aged adults.

## INTRODUCTION

Complaints of difficulty in understanding speech in noisy environments are common, even in individuals whose hearing sensitivity is within the normal ranges^(1-4)^. While threshold pure tone audiometry is considered the gold standard for identifying hearing loss, its results do not reflect the actual listening conditions for speech sounds^(2,3)^.

Several hypotheses have been proposed to account for self-reported hearing difficulties in people with normal hearing. One is the deafferentiation between cochlear nerve fibers and sensory cells, known as cochlear synaptopathy or “hidden hearing loss”, which is undetectable because it does not raise auditory thresholds^(5)^. Studies claim that this promotes poor speech discrimination since it is associated with dysfunction of the medial olivocochlear system, which plays a role in auditory recognition in the presence of noise^(5,6)^. Another hypothesis is that neural alterations involving the central auditory nervous system (CANS) and auditory processing disorder (APD) can occur even in the absence of structural alterations in the CANS^(3)^.

In a study by Hind et al.^(2)^, 4% of adults referred for audiological assessments for complaints of difficulty in understanding speech in noise presented audiograms within normal limits. No other evaluations were performed in these studies to verify the patients’ complaints. It is, however, important to investigate not only the occurrence of such complaints, but also the tools that may be used for audiological assessments, to clarify the phenomenon itself and propose the best strategy. The elucidation of how the self-perception of hearing difficulties is related to speech recognition, not just to auditory sensitivities across different decades of life, should be considered the starting point for such investigations.

This study was thus conducted to investigate the occurrence of speech intelligibility complaints in the presence of noise in subjects with normal hearing. In addition, to compare the occurrence of complaints with performance on a speech-in-noise test, age was used as a variable for both measures.

## METHODS

This observational, cross-sectional study was approved by the Research Ethics Committee of the Faculty of Medicine of Ribeirão Preto, University of São Paulo (no: 2,816,793). All participants signed an informed consent form.

### Participants

Participants were recruited among students and employees of the University and Hospital of São Paulo and in the general community through the researchers' social contacts and dissemination of information.

Inclusion criteria were age between 18 and 59 years, absence of hearing loss, and no diseases suggestive of impairment of the central nervous system (e.g., epilepsy, seizures, migraine), neither currently nor in the past. Exclusion criteria (applied on the day of the hearing assessment) were hearing loss of any nature, altered results on behavioral auditory processing tests (even on one single ear and/or test), and a mental consciousness score lower than that stipulated for the patient’s years of schooling.

The sample consisted of 39 healthy adults of both sexes, divided into four groups (G) according to age. G18 consisted of subjects aged between 18 and 29 years (mean=21.2 years), G30 of those between 30 and 39 years (mean=33.3), G40 of those between 40 and 49 years (mean=43.7), and G50 of those between 50 and 59 years (mean=52.7). G18 and G30 consisted of 10 subjects (four males), G40 of 10 subjects (three males), and G50 of nine subjects (two males).

### Procedures

All 39 subjects underwent a) meatoscopy, b) pure tone audiometry, c) tympanometry, d) behavioral tests of dichotic listening, frequency patterns, and speech in noise, e) a screening of their state of mental consciousness, and f) an auditory self-report.

*Hearing sensitivity:* Pure tone thresholds were investigated with an Otometrics audiometer (model MEDSEN Astera2) and HDA 300 headphones, at frequencies from 0.25 to 8 kHz. Hearing loss was defined as an average hearing loss (0.5, 1, 2, and 4 kHz) ≥25 dB HL. Tympanometry was performed using Otometrics equipment (model ZODIAC 901) with a 226-Hz probe. Compliance values between 0.3 and 1.7 ml obtained between +50 and -150 daPa were considered adequate.

*Dichotic listening test and frequency patterns:* The following tests were chosen because they are sensitive enough to identify CANS dysfunctions^(7)^ and minimize biases. The Brazilian Portuguese version of the Dichotic Digit Test was performed in the binaural integration stage according to the guidelines for application and analysis^(8)^. Scores of ≥95% in both ears were considered normal. The adult version of the Frequency Pattern Test^(9)^ was applied binaurally in the naming stage. The normal value adopted was a score ≥76%.

*Mental awareness:* The Mini-Mental State Examination was also performed in an attempt to minimize other possible biases, which influence the analysis of auditory self-reports and speech perception in noise tests. A score equal to or greater than the cutoff point corresponding to years of schooling was considered normal, according to the translated version (validated for Brazilian Portuguese)^(10)^.

*Auditory self-perception:* The Amsterdam Inventory for Auditory Disability and Handicap (Pt-AIADH)^(11)^ is composed of the auditory domains of detection, location, discrimination and recognition, intelligibility in silence, and intelligibility in noise. Thirty questions are given scores of 03, 02, 01, and 00, referring to the answers “hardly ever”, “sometimes”, “almost always” and “always”, respectively. The results are interpreted as the sum of the answers for each of the domains, with the highest score reflecting the greatest difficulty in auditory activities of daily living (except for questions 18 and 30, where a higher score indicates less hearing difficulty).

*Speech-in-noise test:* The Portuguese Sentence Lists (LSP)^(12)^ test was used, applied in free-field condition, to assess speech perception in noise. The test and analysis were carried out as recommended, in the following steps: a) measurement of noise output level and target signal in the sound box; b) training in silence and noise with list 1A; c) sentence recognition in silence with list 2A; and d) noise recognition with list 2B. The subjects sat 1 m from the amplification box, individually positioned such that the box height corresponded to the height of the individual’s ears, with the degree of incidence of the target signal and noise at 0° azimuth, representing the most unfavorable listening condition. The sentence recognition threshold in noise was determined using the descending-ascending technique, with the noise fixed at 65 dB (A) and the speech initially at an S/N ratio of 0 dB, with an increase or decrease in intensity of 4 dB from the hit or miss and 2 dB after changing the response type. The S/N ratio was established based on the recognition of 50% of the sentences in the noisy condition. The S/N ratio was entered into our analyses as the dependent variable.

### Statistical analyses

Initially, the auditory thresholds of each ear were compared within the same age group using Student’s t-tests for paired samples. The Pt-AIADH and LSP scores were individually assessed according to age group using one-way analysis of variance (ANOVA) with p<0.05; Tukey’s and Dunnett's multiple comparison tests were also applied. Pearson's correlation test was used to assess the variables of the Pt-AIADH and LSP scores in noise. The significance level was set at 5%.

## RESULTS

### Hearing sensitivity

**Figure 1:**
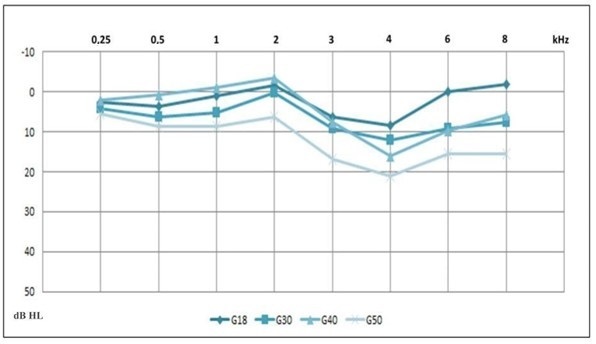
Average of pure tone thresholds, by frequency, according to age group

While all participants had thresholds within the normal range, a comparison was performed between the ears in each group but yielded no statistical differences (p>0.05). This allowed us to compare thresholds between age groups, as a function of the number of ears rather than subjects. Figure 1 shows the differences for all frequencies (except for 0.25 kHz).

### Auditory self-perception

Differences were observed between age groups in the Pt-AIADH speech intelligibility domain score (p<0.05), between G18 and G40 (p=0.0099*; CI=-4.330--0.470), G18 and G50 (p=0.0036*; CI=-4.727--0.760), and G30 and G50 (p=0.0412; CI=-4.020--0.060). In the domain of speech intelligibility in silence, the data showed a tendency towards a significant difference between G18 and G40 (p=0.0662; CI=-2.049-0.4859). In the other three domains, the differences between age groups were not significant (p>0.05) (Table 1).

**Table 1:** Scores of the AIADH-pt domains and the LSP test for each group

**Domains**	**Groups**					**Statistical tests**	
	**G18**	**G30**	**G40**		**G50**		
	**Mean**					**ANOVA**	
	**Minimum-maximum**						
**Detection**	0.7	0.8		0.9	0.6	0.8252	
	0 -2	0-4		0-2	0-3	F (3.35)=0.299	
**Localization**	0.4	1.0		1.3	0.4	0.2035	
	0 -2	0-3		0-4	0-2	F(3.35)=1.615	
**Discrimination-**	0.6	1.0		1.8	0.4	0.2528	
**recognition**	0 -2	0-4		0-4	0-1	F(3.35)=1.422	
**Speech intelligibility in silence**	0.1	0.1		1.0	0.7	0.0354*	
	0 -1	0-1		0-4	0-2	F(3.35)=3.191	
**Speech intelligibility in noise**	0.7	1.4		3.1	3.4	0.0012*	
	0-4	0-3		0-4	0-6	F(3.35)=6.612	
**Total**	2.1	4.3		7.2	5.1	0.1057	
	0-6	01/dez		0-20	0-10	F(3.35)=2.198	
**LSP**	**G18**	**G30**		**G40**	**G50**	**ANOVA**	*Dunn post-hoc*
**S/N ratio**							
**Mean**	-7.8	-2.8		-3.7	-3.2	p=0.0002* F(3.35) =8.69	G18>G30 *p*=0.0007* -8.81 to -2.14
**Minimum**	-12.4	-9.2		-6.8	-5.7		G18>G40 *p*=0.0002* -8.39 to -1.53
**Maximum**	-0.7	2		5	-0.6		G18>G50 *p*=0.0010* -9.01 t -1.96

* Significant values (p≤0.05): one-way ANOVA, and post-hoc Tukey’s multiple comparisons test

**Caption: F** = analysis of variance; **S/N =** signal-to-noise; **LSP =** Portuguese Sentence List; **G18 =** age group from 18 to 29 years; **G30** = age group from 30 to 39 years; **G40 =** age group from 40 to 49 years; **G50 =** age group from 50 to 59 years

### Speech perception in noise

The results of the speech-in-noise test were subjected to a comparative analysis of the silence and noise conditions. Thus, this result shows the S/N ratio obtained between the two moments. The analysis yielded differences in the S/N ratio between groups (p=0.0002*/ F(3.35)=8.69), between G18 and G30 (p=0.0007*/-8.81--2.14), G18 and G40 (p=0.0002*-8.39--1.53), and G18 and G50 (p=0.0010*/-9.01--1.96), with higher S ratio values/R. There were no statistically significant differences among the other groups (Table 1).

### Correlations between variables

Possible relationships between variables with significant results (p<0.05) were further verified. The intelligibility in noise domain scores (Pt-AIADH, and S/N ratio of the LSP test) showed a moderate (r=0.4359) and significant (p=0.0310; CI=0.03-0.59) correlation.

## DISCUSSION

The exclusion criteria applied in the present casuistry study, such as altered results on auditory processing behavioral tests and an altered mental state of consciousness, are important as they minimize the possibility of biases that may interfere with the results of the studied variables^(4,7)^.

### Auditory sensitivity, auditory self-perception, and speech perception

Studies have reported that difficulties in understanding speech in acoustically unfavorable environments are frequent in individuals with normal audiograms^(1-4)^. In the present study, all subjects had adequate hearing sensitivity but complained of hearing difficulties, and all groups scored high on the Pt-AIADH domains and highest on the domain of intelligibility in noise. Davis et al.^(1)^ reported that out of 26% of adults with complaints of difficulties in speech understanding in noise, 10% had tonal thresholds within the normal range. Decades later, other studies corroborated these results^(2-4)^. Possible explanations for these findings include alterations and/or cochlear and neural impairments^(3)^. Cochlear synaptopathy or “hidden hearing loss”, for example, occurs before the loss of sensory cells, which may lead to difficulty in understanding speech in noise^(5,6)^; causes may be exposure to noise, aging, and ototoxic drugs^(5,6)^. At the intra-axial level, studies have questioned the possibility of the presence of TPA in these cases^(2)^. Hind et al.^(2)^ identified the prevalence of this disorder at 0.5 to 1% in this population.

According to Zanchetta et al.^(11)^, there is a relationship between the total Pt-AIADH score and audiological clinical assessment findings. In the current study, we identified no statistical differences in the average inventory scores; however, the trend towards significance in relation to the responses of the groups is noticeable, which can be justified by the number of cases and identified with its increase.

This study reinforces the notion that some aspects are not reflected or identified in the audiogram. Patients in which this is the case usually have no indication for any type of intervention, which generates concern, as they may present with undiagnosed alterations (e.g., TPA) that are subject to remediation^(2)^.

The inclusion of tests that assess the function related to the patient’s complaint would be an important step towards understanding the patient's self-report; ideally, measurements should be conducted with an instrument validated for the intended function.

In our study, the domains that showed significance were intelligibility in silence and noise, with a clear differentiation across groups. The lowest score obtained for the domain of intelligibility in noise in G18 indicates less hearing difficulty and justifies the higher S/N ratio, that is, adequate speech perception in more unfavorable listening conditions compared to the other groups. The need for a lower S/N ratio for older age groups to obtain recognition of 50% of sentences is also reflected in the higher score obtained on the Pt-AIADH for this domain. Although studies have reported that a sharp decline in speech intelligibility occurs in populations over 50 years of age^(13)^, it is possible to infer, based on our results, that from the age of 30, there are subtle, self-perceived changes in auditory performance in activities of daily living that are not identified in assessments of auditory sensitivity^(14,15)^.

Our results of a moderate and significant correlation between the domain of intelligibility in noise and the test that evaluates this function reinforce that self-perception is a subjective measure of hearing that can infer and guide necessary supplementary evaluations.

### Comparison of groups according to age

We observed changes in the three aspects studied with increasing age. Our analysis of auditory sensitivity revealed differences between the groups according to age, with an increase in pure tone thresholds in older individuals. This finding justifies the Pt-AIADH result of greater hearing difficulty in G40 and G50 due to intelligibility in noise. Previous studies have identified that differences in tonal thresholds, even within normal limits, are reflected in complaints of difficulty in understanding speech in noise^(15)^.

The present study has several limitations, and caution is therefore needed in the interpretation and generalization of the results. Although two behavioral tests of auditory processing that are sensitive for the identification of APD have been applied, it is not possible to rule out its presence. In addition, noise exposure and the use of ototoxic drugs were not controlled; thus, the phenomenon of cochlear synaptopathy and/or dysfunction at the brainstem level cannot be ruled out.

These results represent the first step in the investigation of a population of people with normal hearing who complain of difficulty in understanding speech. The next steps concern an increase in sample size and the inclusion of tests that assess other mechanisms and/or abilities of auditory processing to better understand the listening abilities of these individuals.

## CONCLUSION

In the present study, people with normal hearing, and especially older individuals, self-reported difficulty speaking in the presence of noise. We also found that the greater an individual’s difficulty, the worse their performance on the speech-in-noise test; this finding was more significant in middle-aged adults.
