# Supplementary material for: Self-reported hearing difficulties and speech-in-noise test performance - what can we find behind a “normal” audiogram?
Source: Codas. 2023 Nov 27;35(6):e20220111. doi: 10.1590/2317-1782/20232022111p (PMC10702713; doi:10.1590/2317-1782/20232022111p)
Supplement: Translation

# Translation: Autorrelato de dificuldades auditivas e desempenho em teste de fala com ruído - o que podemos encontrar por trás de um audiograma “normal”?

**DOI:** 10.1590/2317-1782/20232022111pt

**Authors:** Pamela Papile Lunardelo, Laura Caetano Meneghelli, Sthella Zanchetta

**Affiliations:** 1 Faculdade de Filosofia, Ciências e Letras de Ribeirão Preto - FFCLRP, Universidade de São Paulo - USP - Ribeirão Preto (SP), Brasil.; 2 Faculdade de Medicina de Ribeirão Preto - FMRP, Universidade de São Paulo - USP - Ribeirão Preto (SP), Brasil.

**Keywords:** Percepção Auditiva, Percepção de Fala, Relação Sinal-Ruído, Inquéritos e Questionários, Adulto

## Abstract

**Objetivo:**

Investigar a queixa de dificuldade de inteligibilidade na presença de ruído, em sujeitos sem perda auditiva e compará-la com o desempenho em um teste de fala no ruído.

**Método:**

A casuística foi constituída por 39 sujeitos de 18 a 59 anos e 11 meses foram divididos em quatro grupos em função da década de vida. Estes foram submetidos à audiometria, timpanometria, testes de processamento auditivo, mini exame do estado mental, autorrelato da percepção auditiva com o Pt-AIADH e a um teste de sentenças no silêncio e no ruído.

**Resultados:**

Todos os grupos pontuaram para os domínios do Pt-AIADH, com maior pontuação média para o domínio de inteligibilidade no ruído. Houve diferenças entre o G18 vs G40, G18 vs G50, e G30 vs G50 para a autopercepção auditiva na inteligibilidade no ruído; e diferenças entre o G18 e os demais grupos no teste de fala com ruído, com menor relação sinal-ruído para os adultos mais velhos. Houve correlação moderada e significativa para a inteligibilidade no ruído e o teste de fala no ruído.

**Conclusão:**

Normo-ouvintes de todas as faixas etárias estudadas apresentaram queixa de inteligibilidade no ruído. Quanto maior a dificuldade auditiva neste domínio pior o desempenho no teste de fala com ruído, sendo mais significativo em adultos de meia idade.

## INTRODUÇÃO

A queixa de dificuldade de compreensão de fala em ambiente ruidoso é comum, inclusive em indivíduos com sensibilidade auditiva dentro dos padrões de normalidade^(1-4)^. “A audiometria tonal limiar é considerada o teste padrão ouro para identificação de perdas auditivas, contudo, seu resultado não reflete a condição real de escuta para os sons de fala^(2,3)^.”

Existem hipóteses para o autorrelato de dificuldade de escuta por normo-ouvintes. Uma delas é a desaferenciação entre as fibras do nervo coclear e das células sensoriais, conhecida como sinaptopatia coclear ou “perda auditiva oculta”, indetectável por não elevar os limiares auditivos^(5)^. Estudos afirmam que esta promove uma pobre discriminação de fala, uma vez associada a uma disfunção do sistema olivococlear medial que tem papel no reconhecimento auditivo na presença de ruído^(5,6)^. Outra hipótese é a alteração neural envolvendo o Sistema Nervoso Auditivo Central (SNAC), o transtorno do processamento auditivo (TPA) pode ocorrer inclusive na ausência de alterações estruturais do SNAC^(3)^.

No estudo de Hind et al.^(2)^ 4% dos adultos encaminhados à avaliação audiológica, com queixa de dificuldade de compreensão de fala no ruído, apresentaram audiograma dentro dos valores de normalidade. No respectivo estudo não foram realizadas outras avaliações com objetivo de esclarecer a queixa. É importante investigar não somente sua ocorrência, mas também, as ferramentas que possam vir a ser utilizadas na avaliação audiológica com objetivo de esclarecer o “fenômeno” e propor a melhor conduta. A elucidação de como a autopercepção de dificuldades auditivas relaciona-se com a tarefa de reconhecimento de fala, e não apenas com a sensibilidade auditiva nas diferentes décadas da vida, deve ser considerada um ponto de partida.

Este estudo foi conduzido para investigar a ocorrência da queixa de dificuldade de inteligibilidade de fala na presença de ruído, em sujeitos normo-ouvintes. Ainda, comparar a queixa com o desempenho em um teste de fala no ruído, tendo a idade como variável para ambas as medidas.

## MÉTODO

Estudo observacional, transversal realizado após aprovação do Comitê de Ética em Pesquisa da Faculdade de Medicina de Ribeirão Preto, Universidade de São Paulo (nº 2.816.793). Todos os sujeitos que participaram do estudo assinaram o termo de consentimento livre e esclarecido.

### Casuística

Os participantes foram recrutados nos espaços intra e extramuros da Universidade e do Hospital, por meio de contato social das pesquisadoras e divulgação entre alunos e funcionários.

Os critérios de inclusão foram idade entre 18 a 59 anos, a ausência de perda auditiva e de doenças sugestivas de comprometimento do Sistema Nervoso Central (e.g. epilepsia, convulsões e migrânea), atuais ou antecedentes. Os critérios de exclusão foram aplicados no dia da avaliação auditiva, a saber, identificação de perda auditiva de qualquer natureza, resultado alterado nos testes comportamentais de processamento auditivo, mesmo que em uma única orelha e/ou teste e escore inferior ao estipulado para os anos de escolaridade, na triagem do estado de consciência mental.

A amostra foi constituída por 39 adultos hígidos, de ambos os sexos, distribuídos em quatro grupos (G) em função da faixa etária. O G18 constituído por sujeitos entre 18 a 29 anos (média=21,2 anos); o G30 entre 30 a 39 anos (média=33,3 anos); o G40 de 40 a 49 anos (média=43,7); e G50 de 50 a 59 anos (média=52,7). O G18 e G30 foram constituídos por 10 sujeitos, sendo 04 do sexo masculino e 06 do feminino; o G40 por 10 sujeitos, 03 do sexo masculino e 07 do feminino; e o G50 por 09 sujeitos, 02 do sexo masculino e 07 do feminino.

### Procedimentos

Todos os 39 sujeitos realizaram: a) meatoscopia b) audiometria tonal; c) timpanometria; d) testes comportamentais de escuta dicótica, padrão de frequência e fala no ruído; e) triagem do estado de consciência mental; f) autorrelato auditivo.

Sensibilidade auditiva - os limiares tonais foram pesquisados com audiômetro da marca Otometrics, modelo MEDSEN Astera2, fone HDA 300, nas frequências de 0.25 a 8 kHz. Considerou-se presença de perda auditiva quando a média (0.5, 1, 2 e 4 kHz) foi ≥ 25 dB NA. A timpanometria foi realizada com o equipamento da marca Otometrics, modelo ZODIAC 901, com sonda de 226Hz. Considerou-se como resultados adequados os valores de complacência entre 0,3 a 1,7 ml obtidos entre +50 a -150 daPA.

Teste de escuta dicótica e padrão de frequência - os testes foram escolhidos por serem sensíveis para identificar disfunções do SNAC^(7)^, minimizando a presença de vieses. O Teste Dicótico de Dígitos, versão português brasileiro, foi realizado em etapa de integração binaural, segundo as orientações de aplicação e análise do manual^(8)^. Considerou-se como normalidade os escores ≥ 95% em ambas as orelhas. O Teste Padrão de Frequência^(9)^, versão adulto, foi aplicado em etapa de nomeação,de forma binaural. O valor de normalidade adotado foi o escore ≥ 76%.

Estado de Consciência mental - o mini exame de consciência mental também foi realizado na perspectiva de minimizar outro possível viés, que influencia na análise do autorrelato auditivo e da percepção de fala no ruído. Considerou-se normalidade o escore igual ou superior ao ponto de corte respectivo aos anos de escolaridade, segundo a proposta traduzida e validada para o português brasileiro^(10)^.

Autopercepção auditiva - o inventário *Amsterdam Inventory for Auditory Disability and Handicap -* Pt-AIADH^(11)^ é composto pelos domínios auditivos de detecção, localização, discriminação e reconhecimento, inteligibilidade no silêncio e inteligibilidade no ruído. São 30 questões pontuadas em 03, 02, 01 e 00 de acordo com as respostas “quase nunca”, “às vezes”, “quase sempre” e “sempre”, respectivamente. Interpreta-se os resultados pela soma das respostas para cada um dos domínios e em seu total, o maior escore obtido reflete a maior dificuldade em atividades auditivas de vida diária, exceto nas questões 18 e 30, que a pontuação superior indica menor dificuldade auditiva.

Teste de fala no ruído - utilizou-se o teste Listas de Sentenças em Português (LSP)^(12)^, aplicado em campo livre, para avaliar a percepção de fala no ruído. A aplicação do teste e análise dos resultados foram conduzidas conforme preconizado, nas seguintes etapas: a) medição do nível de saída do ruído e sinal alvo na caixa sonora; b) treino no silêncio e no ruído com a lista 1A; c) reconhecimento de sentenças no silêncio com a lista 2A; d) reconhecimento no ruído com lista 2B. Os sujeitos sentaram a um metro da caixa de amplificação, posicionada individualmente para que sua altura correspondesse à altura das orelhas dos mesmos, com grau de incidência do sinal alvo e do ruído em 0° azimute, representando a condição de escuta mais desfavorável. O Limiar de Reconhecimento de Sentenças no Ruído foi determinado por meio de técnica descendente-ascendente, com o ruído fixo em de 65 dB (A) e a fala inicialmente com relação S/R de 0 dB, com aumento ou diminuição da intensidade em 4 dB a partir do acerto ou erro e de 2 dB após mudança no tipo de resposta. A relação S/R foi estabelecida a partir do reconhecimento de 50% de sentenças na condição do ruído. Para o presente estudo foi determinada como variável dependente o valor da relação S/R.

### Estatística

Inicialmente analisou-se comparativamente os limiares auditivos de cada uma das orelhas, dentro da mesma faixa etária, com o teste *t-student,* para amostra pareada. Estudou-se individualmente os escores do Pt-AIADH e da LSP em função das faixas etárias por meio da análise de variância Anova *oneway;* havendo p<0,05 utilizou-se o teste *pós-hoc* de *Turkey’s multiple* e *Dunnett’s multiple comparisons test*. Utilizou-se o teste de correlação de Pearson para analisar as variáveis escores do Pt-AIADH e do LSP no ruído. Estabeleceu-se o nível de significância em 5%.

## RESULTADOS

### Sensibilidade auditiva

Mesmo que todos os participantes tenham apresentado limiares dentro da normalidade realizou-se uma comparação dos limiares entre as orelhas, em cada um dos grupos, na qual o resultado mostrou que não houve diferenças estatísticas (p >0,05). Este resultado possibilitou que a comparação dos limiares, entre as faixas etárias, fosse conduzida em função do número de orelhas, não de sujeitos. Na Figura 1 estão os resultados, que mostram as diferenças para todas as frequências, exceto em 0,25 kHz.

**Figura 1:** Média dos limiares tonais, por frequências, em função da faixa etária

### Autopercepção auditiva

Foram observadas diferenças entre as faixas etárias no escore do domínio inteligibilidade de fala no ruído do Pt-AIADH (p <0,05), sendo as diferenças entre o G18 vs. G40 (p=0,0099*; IC= -4,330 a -0,470), G18 vs. G50 (p=0,0036*; IC= -4,727 a -0,760) e G30 vs. G50 (p=0,0412*; IC= -4,020 a -0,060). No domínio de inteligibilidade de fala no silêncio, houve uma tendência entre o G18 vs. G40 (p=0,0662; IC=-2,049 a 0,4859). Nos demais três domínios as diferenças entre as faixas etárias não foram significativas (p>0,05) (Tabela 1).

**Tabela 1:** Escores dos domínios do AIADH-pt e do teste LSP, de cada um dos grupos

**Domínios**	**Grupos**					**Testes estatísticos**	
	**G18**	**G30**	**G40**		**G50**		
	**Média**					**Anova**	
	**Mínimo-Máximo**						
**Detecção**	**0,7**	**0,8**		**0,9**	**0,6**	**0,8252**	
	**0-2**	**0-4**		**0-2**	**0-3**	**F (3,35)=0,299**	
**Localização**	**0,4**	**1**		**1,3**	**0,4**	**0,2035**	
	**0-2**	**0-3**		**0-4**	**0-2**	**F(3,35)=1,615**	
**Discriminação-**	**0,6**	**1**		**1,8**	**0,4**	**0,2528**	
**reconhecimento**	**0-2**	**0-4**		**0-4**	**0-1**	**F(3,35)=1422**	
**Inteligibilidade de fala no silêncio**	**0,1**	**0,1**		**1**	**0,7**	**0,0354** *	
	**0-1**	**0-1**		**0-4**	**0-2**	**F(3,35)=3,191**	
**Inteligibilidade de fala no ruído**	**0,7**	**1,4**		**3,1**	**3,4**	0,0012*	
	**0-4**	**0-3**		**0-4**	**0-6**	**F(3,35)=6,612**	
**Total**	**2,1**	**4,3**		**7,2**	**5,1**	**0,1057**	
	**0-6**	**1-12**		**0-20**	**0-10**	**F(3,35)=2,198**	
**LSP**	**G18**	**G30**		**G40**	**G50**	**Anova**	***Pós-hoc Dunn***
**Relação SR**							
**Média**	**-7,8**	**-2,8**		**-3,7**	**-3,2**	p=0,0002* **F(3,35) = 8,69**	**G18>G30**
							*p*=0,0007*
							**-8,81 a -2,14**
							**G18>G40**
							*p*=0,0002*
							**-8,39 a -1,53**
							**G18>G50**
							*p*=0,0010*
**Mínimo**	**-12,4**	**-9,2**		**-6,8**	**-5,7**		**-9,01 a -1,96**
**Máximo**	**-0,7**	**2**		**5**	**-0,6**		

* Valores significativos (p≤0,05) - Anova *oneway*, Pós-hoc de Turkey’s multiple e Dunnett’s multiple comparisons test

Legenda: **F** = análise de variância; **S/R** = Sinal-ruído; **LSP** = Lista de Sentenças em Português; **G18** = Grupo da faixa etária de 18 a 29 anos; **G30** = Grupo da faixa etária de 30 a 39 anos; **G40** = Grupo da faixa etária de 40 a 49 anos; **G50** = Grupo da faixa etária de 50 a 59 anos

### Percepção de fala no ruído

O resultado do teste de fala no ruído é uma análise comparativa das condições do silêncio e do ruído, assim, este resultado evidencia a relação S/R obtida entre os dois momentos. Houve diferença entre os grupos (*p*=0,0002*/ F_(3,35)_=8,69),com diferenças entre o G18 vs G30 (p=0,0007*/-8,81 a-2,14), G18 vs G40 (p=0,0002*-8,39 a -1,53) e G18 vs G50 (p=0,0010*/-9,01 a -1,96), com maiores valores de relação S/R. Entre os demais grupos não houve diferenças estatísticas (Tabela 1).

### Correlação entre as variáveis

Em última análise, verificou-se a possível relação entre as variáveis com resultados significantes (p<0,05). Os escores do domínio de Inteligibilidade no Ruído, do Pt-AIADH e a relação S/R do teste LSP apresentou correlação moderada (r=0,4359) e significante (p=0,0310; IC=0,03 - 0,59).

## DISCUSSÃO

Os procedimentos delineados como critérios de exclusão para a presente casuística como os resultados alterados dos testes comportamentais do processamento auditivo e da triagem do estado de consciência mental, são importantes, pois minimizam as possibilidades de vieses que podem interferir nos resultados das variáveis estudadas^(4,7)^.

### Sensitividade auditiva, autopercepção auditiva e percepção de fala

A literatura afirma que é frequente a queixa de dificuldade de compreender a fala em ambientes acusticamente desfavoráveis em indivíduos com audiogramas normais^(1-4)^. No presente estudo todos os sujeitos apresentaram adequada sensibilidade auditiva, ainda assim, houve queixas de dificuldades auditivas, pois todos os grupos pontuaram nos domínios do Pt-AIADH, sendo o domínio de inteligibilidade no ruído o de maior pontuação. Davis et al.^(1)^ reportaram que de 26% de adultos com queixa de compreensão de fala no ruído, 10% apresentaram limiares tonais dentro da normalidade. Décadas mais tarde outros trabalhos corroboram estes resultados^(2-4)^. Possíveis justificativas para estes achados seriam alterações e/ou prejuízos cocleares e neurais^(3)^. Considerada como uma destas, a sinaptopatia coclear ou “perda auditiva oculta” ocorre antes da perda das células sensoriais, podendo gerar dificuldade de compreensão de fala no ruído^(5,6)^; esta seria decorrente de exposição ao ruído, envelhecimento e até mesmo por drogas ototóxicas^(5,6)^. Em nível intra-axial, estudos questionaram a presença de TPA como possibilidade nestes casos^(2)^; Hind et al.^(2)^ identificaram a prevalência deste transtorno em 0,5 a 1% nesta população^(2)^.

De acordo com Zanchetta et al.^(11)^, há relação entre o escore total do Pt-AIADH e os achados de avaliação clínica audiológica. Neste estudo não houve diferenças estatísticas para os valores médios obtidos no total do inventário, entretanto é notória a tendência à significância frente às respostas dos grupos, o que pode ser justificado pelo número da casuística e identificado com o seu aumento.

O presente estudo reforça a perspectiva de que alguns aspectos não são refletidos e/ou identificados no audiograma. Usualmente estes pacientes não têm indicação de algum tipo de intervenção, essa situação gera uma preocupação, pois estes podem apresentar alterações não diagnosticadas (e.g. TPA) que são passíveis de remediação^(2)^.

A inclusão de testes que avaliem a função referente à queixa seria um passo importante para compreender o autorrelato do paciente, idealmente mensurado com um instrumento validado para a função pretendida.

Em nosso estudo, os domínios que apresentaram significância foram a inteligibilidade no silêncio e no ruído, diferenciando os grupos. A menor pontuação obtida no domínio de inteligibilidade no ruído pelo G18 indica menor dificuldade auditiva e justifica a maior relação S/R, ou seja, a adequada percepção de fala em condições mais desfavoráveis de escuta em comparação aos outros grupos. A necessidade de uma menor relação S/R para faixas etárias superiores obterem o reconhecimento de 50% das sentenças também é consoante a maior pontuação obtida no Pt-AIADH neste domínio. Ainda que estudos relatem que o declínio acentuado da inteligibilidade de fala ocorra em populações acima de 50 anos^(13)^, é possível inferir, fundamentado em nossos resultados, que a partir dos 30 anos de idade existem mudanças no desempenho auditivo em atividades de vida diária, que embora sutis, são auto percebidas e não seriam identificadas em avaliações da sensibilidade auditiva^(14,15)^.

Nossos resultados de correlação moderada e significativa entre o domínio de inteligibilidade no ruído e o teste que avalia esta função, reforçam que a autopercepção é uma medida subjetiva da audição que pode inferir e direcionar quanto às avaliações suplementares necessárias.

### Comparação dos grupos em função da idade

Para os três aspectos estudados houve modificações com o aumento da idade. A análise da sensibilidade auditiva evidencia a diferença entre os grupos em função da faixa etária, com aumento dos limiares tonais para os de maior idade. Este achado justifica o resultado do Pt-AIADH de maior dificuldade auditiva no G40 e G50 para a inteligibilidade no ruído. Estudos anteriores identificaram que a diferença dos limiares tonais, ainda que dentro da normalidade, é refletida na queixa de dificuldade de compreensão de fala no ruído^(15)^.

O presente estudo tem limitações e, portanto, há necessidade de cautela na interpretação e generalização de seus resultados. Embora tenham sido aplicados dois testes comportamentais de processamento auditivo, sensíveis para a identificação de TPA, não é possível descartar sua presença. Além disso, os aspectos de exposição ao ruído e o uso de ototóxicos não foram controlados, assim, também não se pode descartar o fenômeno de sinaptopatia coclear e/ou de uma disfunção em nível de tronco encefálico.

Estes resultados representam o primeiro passo na investigação da população de normo-ouvintes com queixa de dificuldade de compreensão de fala. Os próximos passos dizem respeito ao aumento da casuística e a inclusão de testes que investiguem outros mecanismos e/ou habilidades do processamento auditivo a fim compreender melhor a condição de escuta destes indivíduos.”

## CONCLUSÃO

Os normo-ouvintes do presente estudo apresentaram o autorrelato de dificuldade de fala na presença do ruído, com maior ocorrência no grupo de maior idade. Também foi constatado que quanto maior a dificuldade pior o desempenho no teste de fala com ruído, sendo esta mais significativa em adultos de meia idade.
